# Impact of Monofloral Pollen Diets on the Development of Hypopharyngeal Glands and Modulation of Enzymatic, Non-Enzymatic, and Ionic Biomarker Activities in Selected Fat Body Segments and Hemolymph of *Apis mellifera* Workers

**DOI:** 10.3390/molecules31081315

**Published:** 2026-04-17

**Authors:** Maciej Sylwester Bryś, Krzysztof Olszewski, Bernard Staniec, Patrycja Staniszewska, Aneta Strachecka

**Affiliations:** 1 Department of Invertebrate Ecophysiology and Experimental Biology, University of Life Sciences in Lublin, 20-280 Lublin, Poland; patrycja.staniszewska@up.edu.pl (P.S.); aneta.strachecka@up.edu.pl (A.S.); 2 Subdepartment of Apidology, Institute of Biological Basis of Animal Production, Faculty of Animal Sciences and Bioeconomy, University of Life Sciences in Lublin, 20-950 Lublin, Poland; krzysztof.olszewski@up.edu.pl; 3 Department of Zoology and Nature Protection, Maria Curie-Skłodowska University, 20-033 Lublin, Poland; bernard.staniec@mail.umcs.pl

**Keywords:** nutrition, biochemical pathway, ecophysiology, stressors, honey bee

## Abstract

The homogenization of landscapes and reduction in floral diversity have increasingly led to low diversity in pollen diets for honey bees. In this study, we examined the effects of monofloral pollen diets based on wind-pollinated (*Corylus* sp., *Pinus* sp.) and insect-pollinated plants (*Brassica napus* L., *Phacelia* sp., *Solidago* sp., *Fagopyrum* sp.) on the development of hypopharyngeal glands (HPGs), activities of enzymatic (AST, ALT, ALP, GGTP) and non-enzymatic (urea, uric acid) biomarkers, as well as magnesium, calcium, and phosphorus concentrations in the hemolymph and fat bodies from different locations (tergite 3, 5 and sternite) in worker bees. Even a small (10%) addition of pollen to sugar candy stimulated the development of *acini* compared to the control group, with phacelia, buckwheat, and goldenrod pollen having the strongest effects. The largest *acini* developed in the 14-day-old bees fed with *Phacelia* pollen, whereas the collecting duct diameters were significantly reduced in all the pollen-supplemented groups. Enzymatic biomarker activities were the highest in the hemolymph of newly emerged bees and increased with age across all the tissues, particularly in tergite 5, with the highest activities recorded in the bees fed with insect-pollinated plant pollen. Non-enzymatic parameters and ion concentrations also varied with tissue type and segmental location, generally increasing with age and reaching the highest values in tergite 5. Regardless of the tissue (the fat body vs. hemolymph), the bees fed a diet containing pollen from *Brassica napus*, *Phacelia*, *Solidago*, and *Fagopyrum* had higher concentrations of calcium, magnesium, and phosphorus, while the effects of hazel and pine pollen were less pronounced. These findings suggest that even limited pollen supplementation can positively shape the morphological and biochemical physiology of worker bees. Understanding these relationships is crucial for developing strategies to support bee health under increasing environmental pressures and changing floral availability.

## 1. Introduction

The honey bee is a species with a global distribution, commonly regarded as the most beneficial insect, providing pollination in ecosystems. This an example of a mutualistic interspecies relationship in which plants provide bees with nectar and/or pollen, and in return bees pollinate the plants [1,2]. Unfortunately, the health and condition of honey bees are increasingly affected by a range of factors resulting in environmental transformation due to human activity (monocultures, large-scale farming leading to landscape simplification, soil degradation, agricultural chemicalization, urbanization, etc.) [3,4,5]. This dilemma gave rise to the emergence and continous dynamically development of the field of bee ecophysiology, which focuses on studying the impact of pollen diet quality on the metabolic and physiological functions of these insects [6,7,8,9,10,11,12].

Importantly, due to the ease of managing the honey bee, the metabolomic, physiological, and behavioral mechanisms of *Apis mellifera* L. have been described much more extensively to date than in many other pollinator species [13]. Nevertheless, many gaps remain regarding the specific nutritional requirements of these insects, and a deeper understanding is crucial for reducing the negative impact of stress factors and for a more effective protection of pollinators in a transforming environment [9,14].

The quantity of pollen produced does not translate into its quality. This results from the proportions of amino acids, saturated and unsaturated fatty acids, macro- and microelements, and other biologically active compounds present in pollen [15,16,17]. On the other hand, even artificial diets based on plant proteins or animal proteins, with calculated proportions of biological compounds, especially amino acids, do not fully replace a pollen diet [18]. This results from bioavailability and metabolic activity [13]. However, the quality of pollen available to honey bees is not limited just to the total protein content but is determined by the aforementioned nutrient profile.

Insect-pollinated plants such as *Brassica napus* L. (rapeseed), *Phacelia tanacetifolia* Benth. (phacelia), *Solidago* sp. (goldenrod), or *Fagopyrum esculentum* (buckwheat) are a source of pollen grains that usually contain at least 14% of total crude protein [15,19,20,21]. Importantly, pollen from anemophilous (wind-pollinated) plants, often characterized by a lower total crude protein content compared to entomophilous pollen, may nevertheless provide a unique and valuable contribution to the diet of bees, considering the macro- and microelements contained in them. An example is pine pollen (*Pinus sylvestris* L.), which contains almost twice as much magnesium than the pollen of phacelia or the Asteraceae plants (Table 1). Therefore, it is not only cultivated plants and weeds but also anemophilous plants, including trees, that diversify the diet of pollinating insects [22,23,24,25].

Unbalanced diets, particularly those based on pollen from a single species (monofloral pollen diet), may lead to deficiencies of nutrients, including macro- and microelements. Thus, the availability of anemophilous plants in the landscape can significantly diversify the diet of bees by providing access to microelements that may be missing in the entomophilous pollen. Finally, wind-pollinated species such as hazel (*Corylus* sp.) are among the first plants to produce pollen in early spring in the Central European climate, providing essential resources during the critical period of colony rebuilding and early development [10]. Where floral diversity is available, foragers, which in principle consume very little pollen themselves, exhibit some preference for collecting pollen from particular plants. This relationship has both genetic and environmental bases [13]. However, in terms of insect feeding preferences, the opinions of scientists are not consistent [26,27,28]. This is another argument confirming that the aspect of nutrition is multi-faceted and requires further research.

The availability of nutrients in the form of nectar and floral pollen affects the rate and efficiency of bee metabolism. The basic metabolic pathways of insects are similar to those of vertebrates [29,30]. The carbohydrate pathway plays an essential role, since simple sugars derived from nectar are used to generate energy, for example, for wing muscle activity [31]. However, it is floral pollen that provides proteins and amino acids, which, for instance, affect the TOR signaling pathways, genes, and neurotransmission (leucine, lysine); participate in immune responses (arginine); and play a role in thermoregulation (proline) and the synthesis of hormone precursors (tryptophan), among others [32,33,34]. In addition, fatty acids present in pollen are precursors for the synthesis of pheromones, phospholipids, lipid membranes, and wax [35]. According to Filipiak et al. [36], the stoichiometry of elements translates into the proper development of wild pollinators and honey bee larvae. Furthermore, attention should be drawn to the complexity of metabolic pathways, as they are related to the reactions that transform substrates into products. In contrast, macro- and microelements cannot be synthesized or substituted in the same way as carbohydrates and amino acids [36]. Elements have structural functions and participate in many physiological processes such as respiration, digestion, or maintaining cell turgor. Their ionic form, as K^+^, Mg^2+^, Ca^2+^, Na^+^ cations and Cl^−^ anions, is related to the water balance in the organism [37,38]. The ions present in the hemolymph and tissues of insects maintain osmotic pressure [39]. Since these ions are essential for the proper functioning of cellular membranes, their numbers identified in the fat body also carry physiological significance. Moreover, magnesium, calcium, and phosphorus ions are commonly used as non-enzymatic biochemical markers in the hemolymph.

While ions are essential for maintaining homeostasis, enzymes constitute biochemical markers of the organism’s metabolic activity. Among the group of transaminases are two enzymes: aspartate transaminase (AST) and alanine transaminase (ALT). Their activities are associated with protein and carbohydrate metabolism. Alkaline phosphatase (ALP) belongs to the hydrolase group. The ALP enzyme is an enzymatic biomarker related to the transport of nutrients between the midgut, hemolymph, and fat body in the honey bee [40,41,42]. The gamma-glutamyl transpeptidase enzyme (GGTP) mediates amino acid transport across cell membranes in insects. Moreover, it participates in glutathione metabolism, meaning that GGTP is related to detoxification metabolism and protection against free radicals [43,44]

In order to fulfill specific functions, nutrients from bee pollen must first be digested into simple molecules such as proteins, amino acids, lipids, glucose, and fructose. The substrates pass from the midgut into the hemolymph. This tissue, apart from distributing substrates to the cells, also serves as a reservoir of energy substrates that may be directly used metabolically, while the excess is stored in the cells of the fat body (trophocytes and oenocytes) [10,45,46]. In addition to storage functions, the fat body plays roles in detoxification and also participates in nitrogen metabolism, the final metabolites of which are uric acid and urea. Therefore, the fat body is considered a tissue with significant biosynthetic and metabolic activities [46].

Moreover, there is a strict relationship linking diet quality with physiology, including the production of royal jelly. Physiological changes in the hypopharyngeal glands (HPGs) are primarily dependent on the age and caste [47]. These changes involve the transition from a nurse bee to a forager [48]. As young worker bees develop, until the 13th day they care for the brood and secrete protein-rich jelly [49] which is used to feed queens, larvae, drones, and other workers [47]. In healthy adult workers, the HPGs are paired structures located in the front of the brain between the compound eyes. Each gland consists of an excretory (collecting) duct and about 800 *acini*, within which there are 6 to 20 bicellular units composed of secretory cells and duct cells. The secretion from the large collecting/excretory duct runs toward the lower pharynx and is released into the mandible [50]. High secretory activity of the HPG is associated not only with acinar areas, but also with remodeling of the intracellular canalicular apparatus through which royal jelly is conveyed. In nurse bees, the canaliculus is expanded and structurally supported by prominent actin rings, whereas in foragers gland regression is accompanied by reduced secretory cell size [51]. More broadly, current concepts in insect exocrine gland biology indicate that secretory output is determined not only by the morphometric development of the gland, but also by the physicochemical properties of the secretion and the resistance to flow within its conducting compartments [51,52].

It is known that HPG development depends on protein availability, particularly on nutritious pollen [53]. To assess the degree of HPG development in workers, measurements of lobules, protein concentration in the glands, or head mass are most commonly used [47,53,54,55,56,57,58,59,60,61,62,63]. On the 14th day of life, the lobules of the HPG should be fully developed. In older workers, after the 15th day of age, the glands gradually shrink, and their secretory activity decreases as a result of cell apoptosis, accompanied by a reduction in rough endoplasmic reticulum, reduction in secretory cells, narrowing of duct cell lumen, and inhibition of protein synthesis. As a result, these glands consist of only a few small *acini* in forager bees.

Since it is already known that monofloral pollen diets exert distinct effects on the energetic, antioxidant, and proteolytic metabolism of the hemolymph and fat body, it was decided to further investigate whether these differences are also reflected in other physiological parameters, including the development of hypopharyngeal glands as well as biomarkers. Therefore, the following hypotheses were formulated: even a small addition (10%) of pollen from insect-pollinated plants, provided within the context of a monofloral diet, exerts a stimulatory effect and increases the size of worker HPGs (Hypothesis 1); different types of pollen affect the activity of enzymatic and non-enzymatic biomarkers in the fat body and hemolymph in different ways (Hypothesis 2); and the concentration of magnesium, calcium, and phosphorus ions depends on the location/segmental structure of the fat body (Hypothesis 3). Finally, the objective was to determine whether monofloral pollen diets based on wind-pollinated plant pollen have a greater effect on the development of HPGs, the activity of enzymatic and non-enzymatic biomarkers, and selected elements than a diet based only on sugar candy (Hypothesis 4).

The aim of this study is to determine the effect of monofloral pollen diets, using *Corylus* sp. and *Pinus* sp., as well as the insect-pollinated plants *Brassica napus* L., *Phacelia* sp., *Solidago* sp., and *Fagopyrum* sp., on the morphological parameters of the HPGs; the activities of aspartate transaminases (ASTs), alanine aminotransferases (ALTs), alkaline phosphatases (ALPs), and gamma-glutamyl transferases (GGTPs); and uric acid c, urea, magnesium, calcium, and phosphorus concentrations in the fat body from T3 (tergite 3), T5 (tergite 5), S (sternite) and the hemolymph.

**Table 1:** Mineral content of pollens used in this study [mg/g dry weight].

	Minerals [mg/g Dry Weight]			Literature
	P	Ca	Mg	
*Pinus* sp.	3.06	0.37	1.09	[44]
*Corylus* sp.	No literature data available			
*Brassica napus*	6.76	2.82	1.41	[21]
*Phacelia* sp.	6.04	1.07	0.55	[19]
Asteraceae (*Helianthus* sp.)	2.59	1.72	0.57	[21]
*Fagopyrum* sp.	6.62	2.08	2.80	[21]

## 2. Results

**Figure 1:**
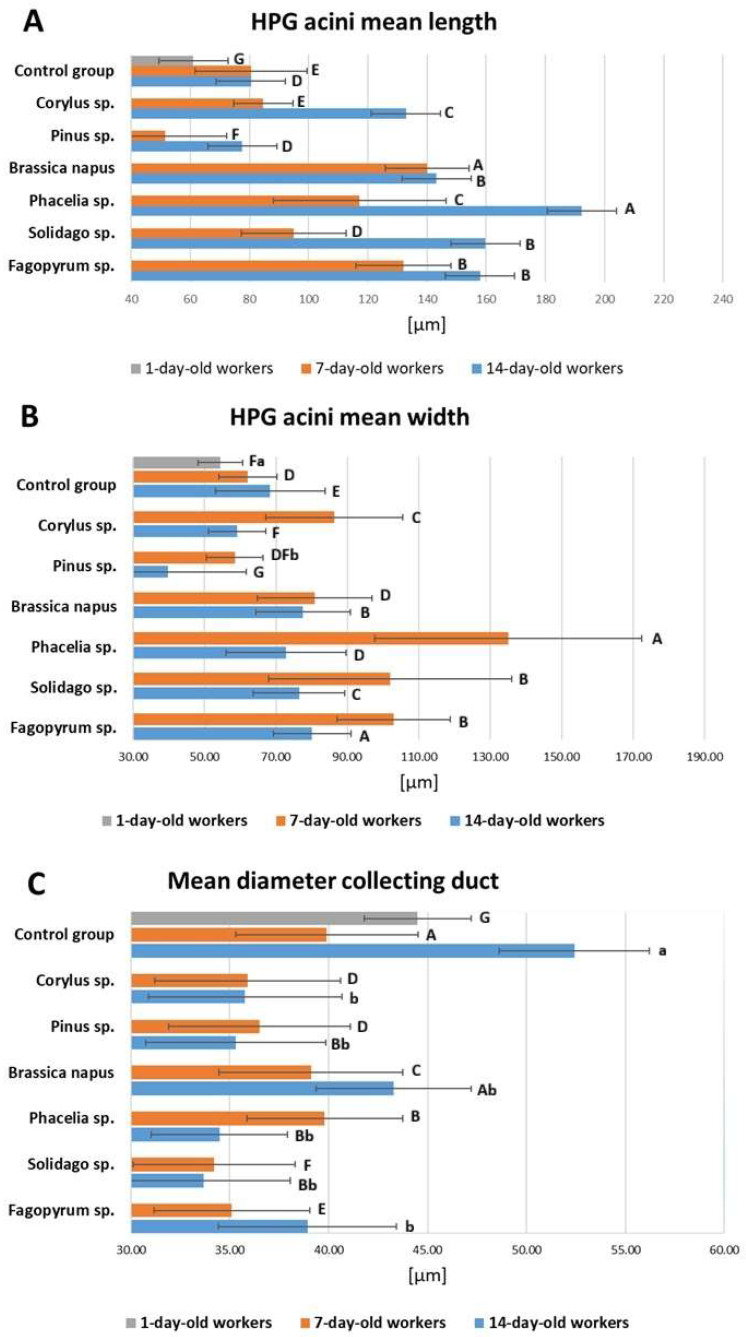
Mean *acini* length (**A**), width (**B**) in the hypopharyngeal glands, and diameter of the collecting duct (**C**) [μm] in 1-, 7-, and 14-day-old workers fed sugar candy (control group) and those fed sugar candy with various types of pollen; *n* = 40. Different lowercase letters indicate statistically significant differences between workers administered pollen and the control ones within identical tissues/sites at *p* ≤ 0.05; different uppercase letters indicate differences significant at *p* ≤ 0.01. Vertical bars indicate standard deviation.

Feeding bees with 10% of pollen added resulted in changes in the length and width of the *acini* (Figure 1). Phacelia, buckwheat and goldenrod pollens promoted the development of *acini*. The opposite effects were observed for the collecting ducts, the diameters of which decreased after bees were fed sugar candy with added pollen. In each group, an age-related increase in the length and width of the *acini* was observed.

In all pollen dietary groups, both in 7-day-old and 14-day-old workers, diet significantly affected acini length and width and the diameter of the collecting duct (Table S1). Moreover, age significantly influenced these parameters across the different dietary groups (Table S2).

### 2.1. Acini Length

The bees fed sugar candy supplemented with pollen tended to have longer *acini* than the worker bees fed candy only (Figure 1A); the bees fed with *Pinus* sp. pollen were the exception. When comparing 7-day-old bees from different groups, the longest *acini* were observed in the group of bees fed with *Brassica napus* pollen (*p* ≤ 0.01). The longest *acini* developed in 14-day-old bees fed with candy supplemented with phacelia pollen (*p* ≤ 0.01).

### 2.2. Acini Width

Overall, *acini* width tended to peak in 7-day-old workers and was lower in 14-day-old workers in most diet groups (Figure 1B). The highest mean width at day 7 occurred in the *Phacelia* group, while among 14-day-old workers relatively larger widths were observed in the *Fagopyrum* and *Solidago* groups.

### 2.3. Diameter of the Collecting Duct

Opposite trends to those in the lengths and widths of *acini* in relation to the effect of feeding with sugar candy with pollen additions were observed in the collecting duct (Figure 1C). The largest collecting duct diameters were observed in the control group and were statistically significantly lower in the groups fed with the 10% pollen supplement (*p*  ≤  0.01). In the case of bees fed sugar candy supplemented with *Corylus*, *Pinus*, *Phacelia*, and *Solidago* pollen, the collecting duct diameter at 14 days of age was significantly smaller than at 7 days of age (*p*  ≤  0.01).

### 2.4. Enzymatic Biomarker Activities

**Figure 2:**
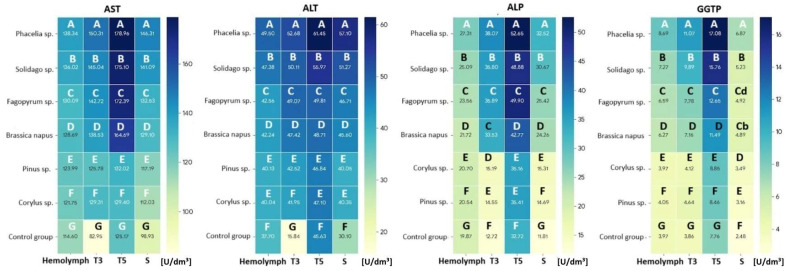
Heatmap of AST, ALT, ALP, and GGTP activities in the hemolymph and fat body from different locations in 7-day-old worker bees fed sugar candy only (control group) and sugar candy with various 10% pollen additions; *n* = 24. Different lowercase letters indicate statistically significant differences among all dietary groups, including pollen-fed workers and the control, within identical tissues/sites at *p* ≤ 0.05; different uppercase letters indicate differences significant at *p* ≤ 0.01.

**Figure 3:**
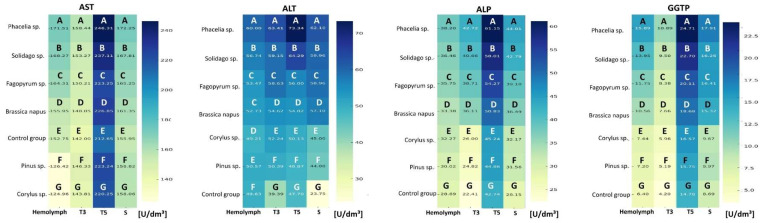
Heatmap of AST, ALT, ALP, and GGTP activities in the hemolymph and fat body from different locations in 14-day-old worker bees fed sugar candy only (control group) and sugar candy with various 10% pollen additions; *n* = 24. Different uppercase letters indicate differences significant at *p* ≤ 0.01.

The activities of biochemical markers AST, ALT, ALP, and GGTP in 1-day-old worker bees were highest in the hemolymph and lowest in the sternite (Table 2). The tissue type and fat body location (T3, T5 and S) significantly affected the activities of these markers in 1-day-old workers (Table S3). Regardless of tissue type and fat body location, their activities increased with age (Table S4; Figure 2 and Figure 3). The addition of pollen to the sugar candy increased marker activities in all tissues compared with the control group, and tissue type (Figure 2 and Figure 3) and fat body location significantly influenced these activities in the different feeding groups (Table S5). Particularly high activities of AST, ALT, ALP, and GGTP were observed in T5 in all groups (*p* = 0.000). The type of diet also significantly affected the activities of these markers in the analyzed tissues (Table S6). Pollen of *Phacelia* and *Solidago* caused the greatest increases in activities compared with the control group in all tissues, especially on day 14 (Figure 3).

### 2.5. Non-Enzymatic Biomarker Activities

**Figure 4:**
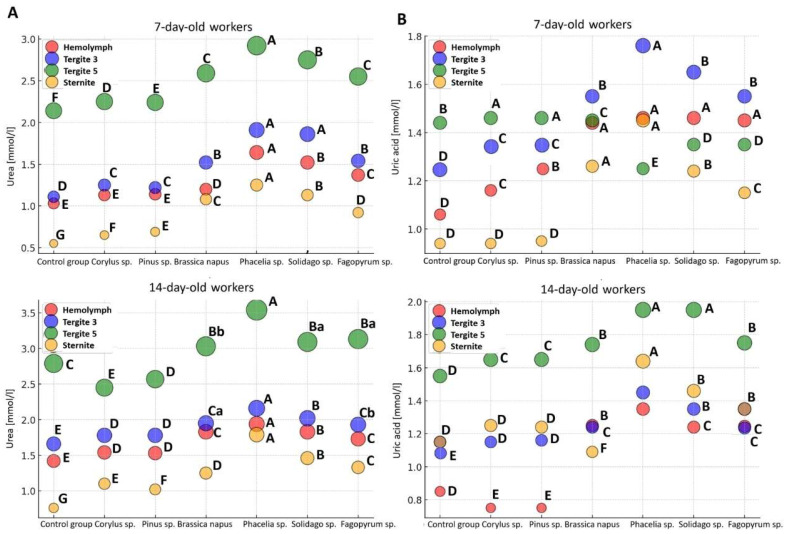
Bubble plots of urea (**A**) and uric acid (**B**) concentrations in the hemolymph and fat body from different locations in 14-day-old worker bees fed sugar candy only (control group) and sugar candy with various 10% pollen additions; *n* = 24. Different lowercase letters indicate statistically significant differences among all dietary groups, including pollen-fed workers and the control, within identical tissues/sites at *p* ≤ 0.05; different uppercase letters indicate differences significant at *p* ≤ 0.01.

The concentrations of non-enzymatic biochemical parameters varied depending on the tissue and the location of the fat body (Figure 4). The concentrations of urea and uric acid were highest in T5 (an exception was uric acid in 7-day-old workers fed sugar candy with *Brassica napus*, *Phacelia* sp., *Solidago* sp., and *Fagopyrum* sp.), while they were lowest in the S (an exception was uric acid in 14-day-old workers).

Age and tissue/location both had significant effects on urea and uric acid concentrations in *A*. *mellifera* L. workers (Tables S7 and S8). Moreover, the effect of tissue/location was statistically significant across all dietary groups in both 7-day-old and 14-day-old workers (Table S8). Diet likewise significantly modified urea and uric acid concentrations in the hemolymph and fat body of workers analyzed at both ages (Table S9).

### 2.6. Ion Concentrations

**Figure 5:**
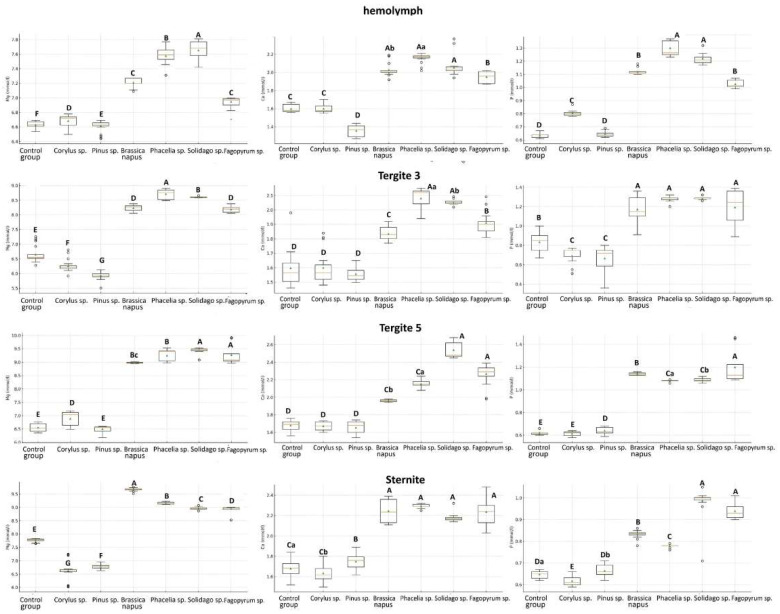
The concentrations of Mg, Ca, and P in the hemolymph and fat body from different locations in 7-day-old worker bees fed sugar candy only (control group) and those fed sugar candy with various 10% pollen additions. The box in the plot represents the interquartile range (IQR) containing the middle 50% of the data, from the first quartile (Q1) to the third quartile (Q3). The line inside the box marks the median value. A triangle inside the box may represent the mean concentration. The scattered dots outside the whiskers are individual data points, often considered outliers; *n* = 24. Different lowercase letters indicate statistically significant differences among all dietary groups, including pollen-fed workers and the control, within identical tissues/sites at *p* ≤ 0.05; different uppercase letters indicate differences significant at *p* ≤ 0.01.

**Figure 6:**
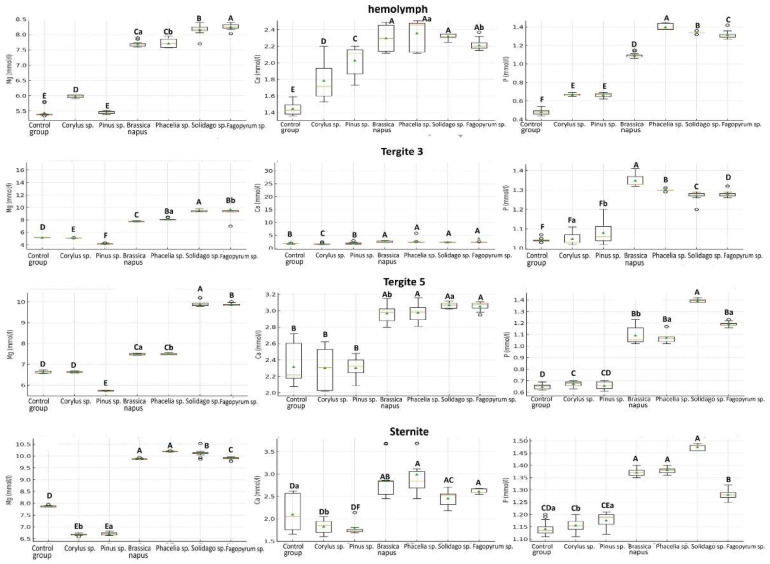
The concentrations of Mg, Ca, and P in the hemolymph and fat body from different locations in 14-day-old worker bees fed sugar candy only (control group) and those fed sugar candy with various 10% pollen additions. The box in the plot represents the interquartile range (IQR) containing the middle 50% of the data, from the first quartile (Q1) to the third quartile (Q3). The line inside the box marks the median value. A triangle inside the box may represent the mean concentration. The scattered dots outside the whiskers are individual data points, often considered outliers; *n* = 24. Different lowercase letters indicate statistically significant differences among all dietary groups, including pollen-fed workers and the control, within identical tissues/sites at *p* ≤ 0.05; different uppercase letters indicate differences significant at *p* ≤ 0.01.

In 1-day-old bees, the highest concentrations of calcium and phosphorus were recorded in the hemolymph, while magnesium was highest in the sternite. Ion concentrations increased with age (Figure 5 and Figure 6). Workers fed sugar candy with a 10% addition of pollen from rapeseed, phacelia, goldenrod, or buckwheat had the highest concentrations of the studied ions, regardless of tissue and age, compared to workers fed sugar candy only. In the case of bees fed with the pollen of wind-pollinated plants (hazel and pine), no clear trends of the studied ions were observed compared to the control group.

With the exception of P concentrations in the hemolymph, age significantly influenced Ca, Mg, and P concentrations in the analyzed tissues/locations of *A*. *mellifera* L. workers (Table S10). For Ca and Mg, significant differences were observed in the hemolymph as well as in the fat body from tergite 3, tergite 5, and sternite. For P, the effect of age was significant in all fat body locations, whereas no statistically significant differences were found in the hemolymph (*p* = 0.104). Both tissue/location (Table S11) and diet (Table S12) significantly influenced ion concentrations in 7-day-old and 14-day-old workers.

## 3. Discussion

Under controlled cage conditions, we fed honey bee workers simulating a monofloral pollen diet. We are aware that this may not reflect full apiary conditions, yet such experiments are commonly used to understand the relationship between nutrition and physiological–biochemical processes [47,56,64,65,66,67,68,69,70,71,72]. In our case, this is the relationship between the monofloral pollen diet and morphological–biochemical changes in HPG tissues, the hemolymph, and the segmental structure of the fat body. Although the honey bee is the subject of many studies, the physiological interrelations of the digestive, circulatory, and immune systems are insufficiently understood [73]. Nutrition is a fundamental process ensuring the growth, development, and maintenance of vital functions [16,74,75]. Nurse bees consume the most pollen and honey in order to produce royal jelly.

In this study, we measured *acini* length and width in both the right and left hypopharyngeal glands. The experiment was performed under laboratory cage conditions to standardize diet exposure, although such conditions may modify the natural division of labor that shapes hypopharyngeal gland development in colonies. Workers were followed until day 14 after emergence to encompass the nurse-age period and early post-nurse stage, when pollen intake and gland activity are typically highest [54,76], and to relate our findings to the canonical nurse-stage peak at 6–9 days and the subsequent regression reported by Crailsheim [77] and Omar et al. [47]. The size of the HPG depends on the age of the honey bee and ranges from 100 μm to over 300 μm [52]. On the 7th day, bees from the group fed a monofloral diet with 10% buckwheat pollen had the longest and widest *acini*. From the literature, it is known that apart from age, the factor determining the development of HPG is the total protein content in pollen. In addition to protein content, HPG development is also influenced by macronutrients, amino acids, and fatty acids. Differences in the proportions of these components are observed between spring and autumn pollen [78]. Buckwheat belongs to cereal plants (although considered a pseudocereal, it is not a member of the family Poaceae), and the protein content in its pollen is only 11.4% [79]. Buckwheat pollen contains amino acids important in the insect diet, such as essential amino acids, e.g., glutamic acid, proline, aspartic acid, leucine, tryptophan, and lysine [21,79]. Such a composition, comprising ten organic compounds, significantly influenced the development and thus the size of the HPG. Phacelia pollen caused elongation and widening of the *acini*, with the highest values observed in 14-day-old bees. This observation clearly confirms our Hypothesis 1, and our results are consistent with other studies indicating that the protein content in insect-pollinated plant pollen affects *acini* development [47,53]. Omar et al. [47] suggested that the consequence of a protein-free, carbohydrate-based diet is smaller HPG *acini* compared to a pollen diet. This observation was also confirmed in our study (see control group). Moreover, we noticed that hazel and pine pollen reduced *acini* size compared to the control. This was a complete surprise to us, as we expected sizes similar to the control group. Both hazel and pine are wind-pollinated plants whose pollen is characterized by low crude protein content [80]. Furthermore, a diet based on a 10% addition of pine pollen to sugar candy increases energy reserves in the form of glucose, glycogen, triglycerides, and protein in the hemolymph and fat body [10]. This is probably due to the fact that pine pollen contains low crude protein concentrations but is rich in microelements, bioactive compounds, and amino acids, which influence biomarkers in bee hemolymph [81]. Further studies are needed to explain the discrepancy between biochemical changes and HPG development under the influence of a monofloral pollen diet. Similarly, as wind-pollinated plants inhibited HPG development, causing smaller *acini*, Ara Begum et al. [52,54] observed that a diet based on maize pollen (*Zea mays* L.) poses a threat to HPG development. Lower concentrations of bioactive compounds such as phenolic acids and antioxidant compounds [80,81] in maize pollen compared to pine pollen may explain the tendencies observed by us and by Ara Begum et al. [54]. The result of small (underdeveloped) *acini* will be limited production and low quality of royal jelly, which will negatively affect the development of the entire colony.

The hypopharyngeal gland consists of *acini* producing jelly, which passes into the collecting duct. The diameters of the collecting ducts range from approx. 33 to 55 µm. These results are consistent with the studies of Klose et al. [82], who determined the diameter of the collecting duct to be 40 µm. Relatively smaller diameters of collecting ducts were recorded in worker groups fed with a 10% pollen addition. Although the addition of phacelia, goldenrod and buckwheat pollen increased the volume of *acini*, it reduced the diameters of the collecting ducts.

In *Apis mellifera*, HPG secretion is produced by secretory cells and conveyed through the duct system to the collecting duct. This conducting pathway is composed of specialized cellular elements in which lumen integrity and secretion transport depend on the organization of duct cells and the F-actin-rich canalicular apparatus. Ultrastructural studies have shown that high secretory activity is associated with expansion of the canalicular system, whereas gland regression is accompanied by thinner canaliculi and reduced secretory cell dimensions, indicating that lumen caliber is under cellular control [80,83,84].

From this perspective, a narrower collecting duct in pollen-fed bees does not necessarily imply impaired gland function. A plausible hypothesis is that protein-rich diets increase biosynthetic investment in *acini*, while the downstream duct system may be functionally adjusted to regulate the residence time and discharge dynamics of a concentrated secretion. Recent work on insect exocrine glands emphasizes that secretion release depends jointly on gland morphology and on the physicochemical properties of the secretion, including viscosity and flow resistance within narrow channels. Thus, if pollen nutrition alters the protein and lipid balance or supramolecular organization of royal jelly precursors, the relationship between acinar size and duct diameter may be non-linear rather than strictly proportional [85].

This interpretation is also consistent with evidence that honey bees can regulate royal jelly production in cage systems and partially buffer nutritional variation in the final secretion, suggesting that secretory output is actively controlled rather than determined solely by gland morphometry [86,87]. At the same time, an artefactual contribution cannot be excluded, because tubular lumina are susceptible to distortion or partial collapse during histological preparation. Consequently, two-dimensional diameter measurements may underestimate the functional lumen if the duct is not perfectly preserved [88].

To fully clarify this phenomenon, future studies should combine morphometric analyses with three-dimensional or ultrastructural imaging, together with direct functional measurements such as gland protein content, major royal jelly protein gene expression level, and royal jelly yield and composition.

As they age, worker bees transition from nursing to foraging for nectar, pollen, and water. As a result of the change in role, dietary preference also shifts, namely, there is a high carbohydrate demand and reduced protein demand. Moreover, the honey bee worker undergoes physiological changes: the hypopharyngeal glands degenerate, the mass of the fat body decreases, the rate of carbohydrate metabolism (mainly glycogenolysis pathways) increases, and the methylation process accelerates [13].

An unbalanced amount of nutrients in the diet may lead to metabolic disorders associated with weakened immune response, reduced body mass, or premature aging [12,52,68,89,90]. It is well known that the activity of enzymatic and detoxification systems in insects, including the honey bee, is strictly dependent on nutrition, age, and the presence of stress factors in the environment [91,92,93,94]. Research expands previous observations concerning the proteolytic and antioxidant systems [70,88], indicating the significant role of enzymatic metabolic markers.

We demonstrated that the activities of AST, ALT, ALP, and GGTP increased with worker age, both in the hemolymph and in the fat body (Hypothesis 2). Differences between fat body segments were distinct—the highest values were recorded in T5, regardless of the type of pollen consumed. This finding contrasts with the results of Strachecka et al. [42] where in 1-day-old bees the highest enzyme activity was noted in T3. The discrepancies may result from the age of insects, the period of conducting the study, as well as the different health conditions of the colonies.

AST and ALT are key transaminases associated with amino acid and carbohydrate metabolism [33]. ALP functions as a hydrolase involved in the transport of nutrients between the midgut, hemolymph, and fat body [40,41]. GGTP, in turn, is responsible for amino acid transport and glutathione metabolism, thus directly participating in detoxification and protection against reactive oxygen [43]. Previous findings by Strachecka’s team and other researchers indicate that increased activity of these markers in the honey bee should be regarded as a positive phenomenon, reflecting intensification of metabolism and adaptive capacity [41,95,96]. In contrast to vertebrates, where high transaminase activity is a biomarker of tissue damage, in bees it may indicate good condition and activation of immune processes [42]. This interpretation is supported by Łoś and Strachecka et al. [41], who showed that in honey bees, elevated activities of these enzymes may accompany physiological activation associated with immune and metabolic responses rather than irreversible tissue damage. The question arises as to whether the increase in enzymatic marker activity induced by a monofloral pollen diet is a positive or negative effect. On the one hand, elevated enzymatic activity is a positive reaction to the presence of certain compounds in floral pollen and acts as an internal protective mechanism against harmful stressors such as pesticides [97], the use of the antibiotic amphotericin B [98], or electromagnetic fields [99], which are known to reduce the activity of these markers. On the other hand, although a monofloral pollen diet works better than a diet based solely on sugar, the latest studies (including our previous work) indicate that a diversified diet is optimal. The increase in enzymatic activity may be interpreted as a physiological cost of adaptation to less than ideal conditions.

However, the interpretation of elevated enzymatic activity exclusively as a beneficial indicator of metabolic activation should be considered with some caution. In insects, sustained increases in metabolic enzyme activities may also reflect elevated metabolic turnover associated with higher energetic costs. In pollinators, accelerated metabolic processes are often linked with increased production of reactive oxygen species and greater physiological wear, potentially contributing to metabolic stress or accelerated aging under certain conditions [100]. From this perspective, higher activities of AST, ALT, ALP, and GGTP observed in pollen-fed bees may reflect both enhanced metabolic performance and increased biochemical turnover associated with intensive nutrient processing. Nevertheless, because our experiment covered only up to 14 days of worker life, it remains unclear whether such enzymatic elevation represents a purely adaptive response or whether it may indicate an accelerated aging process. A possible explanation for the discrepancy between increased metabolic enzyme activity and reduced acinar development may lie in the distinct physiological functions of metabolic and secretory tissues in worker bees. Following digestion in the midgut, pollen-derived nutrients are absorbed into the hemolymph and stored in the fat body for detoxification. Consequently, elevated activities of AST, ALT, ALP, and GGTP may reflect intensified processing and turnover of dietary nutrients rather than increased synthetic royal jelly (RJ). In contrast, the development of the HPGs depends on the availability and balance of specific nutrients required for royal jelly biosynthesis, particularly essential amino acids and lipids [101].

Moreover, differences in enzymatic activities may also be influenced by variation in the digestibility and bioavailability of nutrients from different pollen types [2,16,18,27]. Overall, the physiological response may also depend on the digestibility of particular pollen types, because the pollen wall is a major barrier limiting access to intracellular nutrients in honey bees. For example, Castanea pollen was reported to be digested more efficiently than *Trifolium* pollen, and mechanical crushing of *Zea mays* pollen increased diet digestibility and hemolymph protein content, indicating that nutrient bioavailability depends not only on chemical composition but also on exsine structure [102,103].

Our results revealed that the highest concentrations of urea and uric acid, regardless of the nutritional group, occurred in T5 (Hypothesis 3). Both metabolites are end products of nitrogen metabolism and protein turnover, serving detoxification and antioxidant functions [44,104,105,106]. Uric acid is considered a non-enzymatic antioxidant capable of neutralizing free radicals and protecting proteins against oxidative damage [96,101]. Such properties confirm the thesis that the fat body in insects plays a role analogous to the liver in vertebrates. Interestingly, the high concentration of nitrogen metabolites in T5 correlates with earlier observations indicating the detoxification functions of this segment [42,94,102].

Ions, especially Ca^2+^, Mg^2+^, and P, play a key role in bee metabolism. Research indicates that pollen from insect-pollinated plants provides the most bioavailable forms of these elements (particularly sugar candy with the addition of pollen of phacelia, goldenrod, and buckwheat). Calcium ions modulate the activity of calcium phospholipases and thus influence the humoral immune response [107]. They also participate in muscle contractions responsible for wing movement; moreover, they are cofactors of many metabolic and detoxification enzymes. The development of honey bee larvae depends on the food provided by nurse bees. Magnesium and phosphorus, as well as other macro- and microelements, may be limiting factors [36]. It should also be noted that mineral composition data for *Corylus* sp. pollen were not identified in the available literature.

There is a strong relationship linking bee physiology with different floral resources in the surrounding landscape. Climate change, monocultures, and herbicide use cause temporal mismatches between flowering periods, for example, early-spring species such as hazel, and colony development. Therefore, there is a need, firstly, to develop tools for monitoring changes in flowering phenology, e.g., supported by satellite data combined with artificial intelligence; secondly, to identify nutritional biomarkers in order to link bee physiology with plant habitats; and finally, to develop actions to mitigate the effects and compensate for nutritional shortages in the form of dedicated flower mixtures or sugar candy with pollen addition.

## 4. Materials and Methods

### 4.1. Fresh Pollen Collection and Preparation of Sugar Candy with Various Pollen Additions

Fresh bee pollen was collected using pollen traps placed located at the hives of beekeepers in Poland. Pollen loads were first manually color-sorted as a shortcut to identify the dominant pollen source. Microscopic pollen preparations were then used to confirm the identity of the pollen through morphological features. Samples were examined using an MBL 800 Microscope at 40 × 15 magnification. The sugar candy mix was made from 500 mL water and 2.3 kg of white granulated sugar, and the sugar candy was divided into equal parts/the following groups:

- Control group—no pollen was added, fed sugar candy only;
- Experimental groups—10% of one type of pollen, hazel (*Corylus* sp.), pine (*Pinus* sylvestris L.), rapeseed (*Brassica napus* L.), phacelia (*Phacelia tanacetifolia* Benth), goldenrod (*Solidago* sp.), or buckwheat (*Fagopyrum esculentum* Moench), was added to the remaining groups of sugar candy.

The pollen dose was determined based on our studies and those of other authors [10,12,72,108], taking into account the situation that one worker bee consumes 10 mg of protein and 90 mg of carbohydrates per 100 mg of diet.

### 4.2. Cage Tests

One-day-old bees were obtained from 3 colonies on an apiary belonging to the University of Life Sciences in Lublin (51°22′ N, 22°63′ E), Poland, according to Bryś et al. [10] and Strachecka et al.’s method [46]. A queen from each of these colonies was caged within a queen-excluder comb-cage containing one empty comb for 24 h for the eggs to be laid. After 20 days from the moment the eggs were laid, the brood combs were placed in an incubator (temperature 34.5 °C; relative humidity 60%). Freshly emerged worker bees mixed and were placed in 56 cages (40 workers in each cage) which were divided into 7 groups: a control group fed with sugar candy and six groups fed from the first day with sugar candy with the addition of 10% of specific pollen. The bees were fed ad libitum. At the beginning of the experiment, 40 one-day-old workers were collected for morphological analysis (hypopharyngeal gland measurements). Every two days, dead workers were removed from the cages and candy was supplemented. When the workers were 7 and 14 days old, we collected 40 workers per group and morphological analyses were performed.

Moreover, at the age of 7 and 14 days, living worker bees were collected from the cages for biochemical analyses (*n* = 24 bees per group). This resulted in biochemical analyses of the following dataset: 24 1-day-old workers + 24 worker bees × 7 groups × 2 samplings.

### 4.3. Hypopharyngeal Gland Measurements

The live 7- and 14-day-old workers were randomly selected from each wood cage and immediately decapitated. The head was cut open under a stereoscopic microscope. The glands from the right and left sides of the head were extracted with micro-scissors and placed on a microscope slide in a drop of 0.6% *natrium chloratum* (pro inj.). In every HPG, the lengths and widths of 20 random *acini* were measured using the Camera Olympus DP72 program (Microscope Olympus BX61, Tokyo, Japan; magnification 40×). Moreover, the diameters of the collecting ducts were measured at 20 random points in each HPG.

### 4.4. Hemolymph Collection

A small puncture was made in the abdomen intersegmental membrane using a sterile preparation needle. Hemolymph samples (4 μL per worker) were collected from live worker bees using a sterile glass capillary (without anticoagulant; Medlab Products, Raszyn, Poland) according to the method of Łoś and Strachecka [41]. Each hemolymph sample was immediately transferred into a sterile Eppendorf tube containing 200 μL of ice-cooled 0.6% NaCl solution and stored at −25 °C for subsequent biochemical analyses.

### 4.5. Fat Body Collection

These bees were euthanized by freezing and then gradually thawed before dissection. Under a stereomicroscope, the fat bodies were carefully isolated in a 0.6% NaCl solution from the T3, T5 and S. Each fat body segment was placed in a separate Eppendorf tube (Hamburg, Germany). Based on previous research by [46,88], the fat bodies from the T3, T5 and S were selected for biochemical analyses due to their high metabolic activity compared to other fat body segments.

### 4.6. Sample Preparation for Biochemical Analyses

Each sample was manually homogenized using a hand-held homogenizer. The homogenates were centrifuged at 4 °C for 1 min at 3000× *g* to separate cellular debris. The resulting supernatants were carefully transferred to new sterile Eppendorf tubes and immediately frozen at −24 °C ± 1 °C for subsequent biochemical analyses.

### 4.7. Metabolic Markers

Metabolic biomarker activities and concentrations were assessed in both hemolymph and fat body samples.

### 4.8. Enzymatic Biomarker Activities

The AST, ALT, ALP, and GGTP activities were determined using the commercial kit (used Alpha Diagnostics^®^ agents, Warsaw, Poland) according to the instructions modified by Łoś and Strachecka [41].

### 4.9. Non-Enzymatic Biomarker Activities

Uric acid and urea concentrations were determined using the commercial kit (Alpha Diagnostics^®^, Warsaw, Poland).

### 4.10. Ion Concentration

Calcium, magnesium and phosphorus concentrations were determined using the commercial kit (Alpha Diagnostics^®^, Warsaw, Poland).

### 4.11. Statistical Analysis

Statistical analyses were performed using Statistica 13.3 (TIBCO Software Inc., Palo Alto, CA, USA; StatSoft Inc., Tulsa, OK, USA). Data normality was assessed with the Shapiro–Wilk test. Since the data were not normally distributed, the effects of dietary pollen type on the parameters of the hypopharyngeal glands (*acini* length and width), the activities of enzymatic markers (AST, ALT, ALP, GGTP) and non-enzymatic markers (urea and uric acid), as well as elemental concentrations were evaluated using the Mann–Whitney U test. The activities of enzymatic and non-enzymatic markers and elemental concentrations were analyzed separately for each tissue/location (hemolymph and fat body from tergites 3 and 5 and the sternite). Differences in the activities of enzymatic and non-enzymatic markers and in the ion concentrations between the hemolymph and fat body locations (T3, T5, and S) of 1-day-old *A. mellifera* L. workers were also compared using the Mann–Whitney U test. Results were considered statistically significant at *p* ≤ 0.05 and highly significant at *p* ≤ 0.01.

## 5. Conclusions

It should be remembered that bee condition is shaped primarily by nutrition. Research provides evidence that monofloral pollen diets affect morphological–biochemical parameters differently, taking into account the segmental structure of the fat body. Understanding the complex network of connections between diet, HPG development, hemolymph, and the segmental structure of the fat body is crucial for pollinator protection in times of increasing anthropopressure and climate change, which, among other things, cause shifts in the phenology of plant flowering.

Our findings highlight the need for a deeper exploration of the nutritional physiology of honey bees in the context of global environmental changes. The ongoing simplification of landscapes, decline in floral diversity, and expansion of monocultures contribute to nutritional stress, which may cascade into metabolic dysregulation and impaired immunity. Future research should therefore focus on the integrative understanding of how diet composition interacts with multiple environmental, chemical, and biological stressors to shape bee metabolism and colony immunity. Promising directions include investigating metabolic pathways under cumulative stress, assessing the physiological effects of pollen from invasive or modified plant species, and developing biochemical biomarker panels for rapid detection of sublethal stress in bees.

Such interdisciplinary approaches combining biochemistry, ecophysiology, and emerging analytical technologies will be essential for predicting the physiological consequences of ongoing habitat transformation and for designing effective strategies to sustain pollinator health in a changing world.

## Figures and Tables

**Table 2:** Activities of enzymatic and non-enzymatic markers as well as concentrations of ions in the hemolymph and fat body (tergite 3, tergite 5, or sternite) of 1-day-old *A. mellifera* L. workers.

**Biomarkers Activities/Concentrations**									
**Tissue/Location**	**AST [U/dm^3^]**	**ALT [U/dm^3^]**	**ALP [U/dm^3^]**	**GGTP [U/dm^3^]**	**Urea [mmol/L]**	**Urea Acid [mmol/L]**	**Mg [mmol/L]**	**Ca [mmol/L]**	**P [mmol/L]**
Hemolymph	30.33 A (±3.21)	20.65 A (±5.55)	4.82 A (±0.10)	1.65 A (±0.37)	0.44 B (±0.01)	1.41 A (±0.01)	7.52 B (±1.73)	1.83 A (±0.83)	0.95 A (±0.22)
Tergite 3	5.60 C (±0.01)	6.54 C (±0.05)	0.52 C (±0.01)	0.48 C (±0.02)	0.13 D (±0.01)	0.23 D (±0.01)	6.62 C (±1.22)	1.61 Bb (±0.64)	0.63 B (±0.01)
Tergite 5	10.47 B (±0.01)	7.73 B (±0.11)	1.51 B (±0.23)	0.80 B (±0.01)	0.74 A (±0.01)	0.44 B (±0.01)	6.54 C (±1.73)	1.45 C (±1.13)	0.56 C (±0.01)
Sternite	3.21 D (±0.12)	5.40 D (±0.05)	0.21 D (±0.01)	0.30 D (±0.01)	0.35 C (±0.0)	0.35 C (±0.01)	8.19 A (±2.68)	1.65 Ba (±0.99)	0.58 C (±0.01)

Different uppercase and lowercase letters indicate differences significant at *p* ≤ 0.01; standard deviation is shown in round brackets.

## Data Availability

All relevant data for this study are publicly available from the RepOD repository https://doi.org/10.18150/TSMDSX.

## Supplementary Materials

The following supporting information can be downloaded at: https://www.mdpi.com/article/10.3390/molecules31081315/s1, Table S1: Effect of diet on *acini* parameters (length, width, and diameter of the collecting duct) in different age groups of *A. mellifera* workers (7-day-old and 14-day-old).; Table S2: Effect of age on *acini* parameters (length, width, and diameter of the collecting duct) in *A. mellifera* workers from different dietary groups.; Table S3: Effect of tissue/location (hemolymph and fat body: tergite 3, tergite 5, or sternite) on the activities of enzymatic and non-enzymatic markers and on ion concentrations in 1-day-old *A. mellifera* workers.; Table S4: Effect of age on the activities of enzymatic markers in different tissues/locations (hemolymph and fat body: tergite 3, tergite 5, or sternite) in *A. mellifera* workers.; Table S5: Effect of tissue/location on the activities of enzymatic markers in 7-day-old and 14-day-old *A*. *mellifera* L. workers from different dietary groups.; Table S6: Effect of diet on the activities of enzymatic markers in different tissue/location types in 7-day-old and 14-day-old *A. mellifera* L. workers.; Table S7: Effect of age on urea and uric acid concentrations in the hemolymph and fat body of *A. mellifera* L. workers.; Table S8: Effect of tissue/location (hemolymph and fat body) on urea and uric acid concentrations in 7-day-old and 14-day-old *A. mellifera* L. workers from different dietary groups.; Table S9: Effect of diet on urea and uric acid concentrations in the hemolymph and fat body of 7-day-old and 14-day-old *A. mellifera* L. workers.; Table S10: Effect of age on Mg, Ca, and P concentrations in different tissues/locations of *A. mellifera* L. workers.; Table S11: Effect of diet on hemolymph Mg, Ca, and P concentrations in 7-day-old and 14-day-old *A. mellifera* L. workers from different dietary groups.; Table S12: Effect of diet on Mg, Ca, and P concentrations in the hemolymph of 7-day-old and 14-day-old *A. mellifera* L. workers.
