# Supplementary material for: Impact of Monofloral Pollen Diets on the Development of Hypopharyngeal Glands and Modulation of Enzymatic, Non-Enzymatic, and Ionic Biomarker Activities in Selected Fat Body Segments and Hemolymph of Apis mellifera Workers
Source: Molecules. 2026 Apr 17;31(8):1315. doi: 10.3390/molecules31081315 (PMC13118541; doi:10.3390/molecules31081315)
Supplement: Supplementary file 1 [file molecules-31-01315-s001.zip › molecules-4229850-supplementary.pdf]

Article

Impact of monofloral pollen diets on the development of hypopharyngeal glands and modulation of enzymatic, non-enzymatic, and ionic biomarker activities in selected fat body segments and hemolymph of *Apis mellifera* workers

Supplementary:

Table S1. Effect of diet on *acini* parameters (length, width, and diameter of the collecting duct) in different age groups of *A. mellifera* workers (7-day-old and 14-day-old).

Age group	**Acini length**	**Acini width**	**Dimeter collection duct**
7-day-old workers	H =206.35, p = 0.000 df = 6	H = 117.58, p = 0.000 df = 6	H = 146.84, p = 0.000 df = 6
14-day-old workers	H = 174.47, p = 0.000 df = 6	H = 183.14, p = 0.000 df = 6	H = 60.27, p = 0.000 df = 6

H—value of statistics for the Kruskal–Wallis test;

p—probability value; df—number of degrees of freedom .

Table S2. Effect of age on *acini* parameters (length, width, and diameter of the collecting duct) in *A. mellifera* workers from different dietary groups.

Groups	Acini length	Acini width	Dimeter collection duct
Control gr.	H = 32.01, p = 0.000 df = 2	H = 38.08, p = 0.000 df. 2	H = 58.86, p = 0.000 df. 2
Hazel	H = 82.10, p = 0.000 df = 2	H = 85.59, p = 0.000 df = 2	H = 64.08, p = 0.000 df = 2
Pine	H = 47.16, p = 0.000 df = 2	H = 15.49, p = 0.000 df = 2	H = 67.80, p = 0.000 df = 2
Rape	H = 79.79, p = 0.000 df = 2	H = 67.71, p = 0.000 df = 2	H = 30.45, p = 0.000 df = 2
Phacelia	H = 96.75, p = 0.000 df = 2	H = 88.51, p = 0.000 df = 2	H = 72.87, p = 0.000 df = 2
Goldenrod	H = 88.26, p = 0.000 df = 2	H = 70.57, p = 0.000 df = 2	H = 72.98, p = 0.000 df = 2
Buckwheat	H = 85.60, p = 0.000 df = 2	H = 90.81, p = 0.000 df = 2	H = 64.12, p = 0.000 df = 2

H—value of statistics for the Kruskal–Wallis test; p—probability value; df—number of degrees of freedom.

Table S3. Effect of tissue/location (hemolymph and fat body: tergite 3, tergite 5, or sternite) on the activities of enzymatic and non-enzymatic markers and on ion concentrations in 1-day-old *A. mellifera* workers.

Biomarkers								
**AST**	**ALT**	**ALP**	**GGTP**	**urea**	**urea acid**	**Mg**	**Ca**	**P**
H = 89.07 p = 0.000 df = 3	H = 88.95 p = 0.000 df = 3	H = 89.08 p = 0.000 df = 3	H = 88.54 p = 0.000 df = 3	H = 83.35 p = 0.000 df = 3	H = 89.08 p = 0.000 df = 3	H = 79.19 p = 0.000 df = 3	H = 74.23 p = 0.000 df = 3	H = 62.75 p = 0.000 df = 3

H—value of statistics for the Kruskal–Wallis test; p—probability value; df—number of degrees of freedom.

Table S4. Effect of age on the activities of enzymatic markers in different tissues/locations (hemolymph and fat body: tergite 3, tergite 5, or sternite) in *A. mellifera* workers.

Tissue/location	Biomarkers activities			
	**AST**	**ALT**	**ALP**	**GGTP**
Hemolymph	H = 153.23<br>p = 0.000<br>df = 2	H = 236.56<br>p = 0.000<br>df = 2	H = 267.66<br>p = 0.000<br>df = 2	H = 172.55<br>p = 0.000<br>df = 2
Tergite 3	H = 267.83<br>p = 0.000<br>df = 2	H = 99.66<br>p = 0.000<br>df = 2	H = 109.70<br>p = 0.000<br>df = 2	H = 185.07<br>p = 0.000<br>df = 2
Tergite 5	H = 267.83<br>p = 0.000<br>df = 2	H = 107.02<br>p = 0.000<br>df = 2	H = 128.16<br>p = 0.000<br>df = 2	H = 189.08<br>p = 0.000<br>df = 2
Sternite	H = 215.28<br>p = 0.000<br>df = 2	H = 136.25<br>p = 0.000<br>df = 2	H = 131.96<br>p = 0.000<br>df = 2	H = 164.06<br>p = 0.000<br>df = 2

H—value of statistics for the Kruskal–Wallis test; p—probability value; df—number of degrees of freedom.

Table S5. Effect of tissue/location on the activities of enzymatic markers in 7-day-old and 14-day-old *A. mellifera* L. workers from different dietary groups.

Groups	Biomarkers							
	7-day-old workers				14-day-old workers			
	**AST**	**ALT**	**ALP**	**GGTP**	**AST**	**ALT**	**ALP**	**GGTP**
Control gr.	H = 89.04<br>p = 0.000<br>df = 3	H = 89.08<br>p = 0.000<br>df = 3	H = 83.16<br>p = 0.000<br>df = 3	H = 80.18<br>p = 0.000<br>df = 3	H = 89.07<br>p = 0.000<br>df = 3	H = 89.08<br>p = 0.000<br>df = 3	H = 82.55<br>p = 0.000<br>df = 3	H = 89.08<br>p = 0.000<br>df = 3
Hazel	H = 80.23<br>p = 0.000<br>df = 3	H = 80.71<br>p = 0.000<br>df = 3	H = 80.21<br>p = 0.000<br>df = 3	H = 88.61<br>p = 0.000<br>df = 3	H = 89.07<br>p = 0.000<br>df = 3	H = 88.59<br>p = 0.000<br>df = 3	H = 80.21<br>p = 0.000<br>df = 3	H = 89.09<br>p = 0.000<br>df = 3
Pine	H = 83.53<br>p = 0.000<br>df = 3	H = 80.88<br>p = 0.000<br>df = 3	H = 80.36<br>p = 0.000<br>df = 3	H = 89.08<br>p = 0.000<br>df = 3	H = 89.08<br>p = 0.000<br>df = 3	H = 78.58<br>p = 0.000<br>df = 3	H = 87.04<br>p = 0.000<br>df = 3	H = 89.08<br>p = 0.000<br>df = 3
Rape	H = 40.57<br>p = 0.000<br>df = 3	H = 43.62<br>p = 0.000<br>df = 3	H = 44.08<br>p = 0.000<br>df = 3	H = 44.09<br>p = 0.000<br>df = 3	H = 44.09<br>p = 0.000<br>df = 3	H = 41.27<br>p = 0.000<br>df = 3	H = 40.16<br>p = 0.000<br>df = 3	H = 44.09<br>p = 0.000<br>df = 3
Phacelia	H = 89.07<br>p = 0.000<br>df = 3	H = 89.03<br>p = 0.000<br>df = 3	H = 90.01<br>p = 0.000<br>df = 3	H = 89.08<br>p = 0.000<br>df = 3	H = 81.79<br>p = 0.000<br>df = 3	H = 85.49<br>p = 0.000<br>df = 3	H = 84.44<br>p = 0.000<br>df = 3	H = 89.08<br>p = 0.000<br>df = 3
Goldenrod	H = 88.11<br>p = 0.000<br>df = 3	H = 86.22<br>p = 0.000<br>df = 3	H = 89.07<br>p = 0.000<br>df = 3	H = 89.11<br>p = 0.000<br>df = 3	H = 80.83<br>p = 0.000<br>df = 3	H = 84.59<br>p = 0.000<br>df = 3	H = 88.29<br>p = 0.000<br>df = 3	H = 89.08<br>p = 0.000<br>df = 3
Buckwheat	H = 81.80<br>p = 0.000<br>df = 3	H = 85.91<br>p = 0.000<br>df = 3	H = 89.08<br>p = 0.000<br>df = 3	H = 89.08<br>p = 0.000<br>df = 3	H = 81.01<br>p = 0.000<br>df = 3	H = 80.58<br>p = 0.000<br>df = 3	H = 80.84<br>p = 0.000<br>df = 3	H = 89.10<br>p = 0.000<br>df = 3

H—value of statistics for the Kruskal–Wallis test; p—probability value; df—number of degrees of freedom.

Table S6. Effect of diet on the activities of enzymatic markers in different tissue/location types in 7-day-old and 14-day-old *A. mellifera* L. workers.

Tissue/location	Biomarkers							
	7-day-old workers				14-day-old workers			
	**AST**	**ALT**	**ALP**	**GGTP**	**AST**	**ALT**	**ALP**	**GGTP**
Hemolymph	H = 149.10<br>p = 0.000<br>df = 6	H = 148.24<br>p = 0.000<br>df = 6	H = 146.02<br>p = 0.000<br>df = 6	H = 151.56<br>p = 0.000<br>df = 6	H = 150.89<br>p = 0.000<br>df = 6	H = 145.88<br>p = 0.000<br>df = 6	H = 148.35<br>p = 0.000<br>df = 6	H = 151.56<br>p = 0.000<br>df = 6
Tergite 3	H = 150.12<br>p = 0.000<br>df = 6	H = 147.67<br>p = 0.000<br>df = 6	H = 144.94<br>p = 0.000<br>df = 6	H = 150.88<br>p = 0.000<br>df = 6	H = 147.14<br>p = 0.000<br>df = 6	H = 147.40<br>p = 0.000<br>df = 6	H = 147.26<br>p = 0.000<br>df = 6	H = 150.64<br>p = 0.000<br>df = 6
Tergite 5	H = 151.39<br>p = 0.000<br>df = 6	H = 146.72<br>p = 0.000<br>df = 6	H = 148.12<br>p = 0.000<br>df = 6	H = 151.56<br>p = 0.000<br>df = 6	H = 147.63<br>p = 0.000<br>df = 6	H = 151.53<br>p = 0.000<br>df = 6	H = 147.95<br>p = 0.000<br>df = 6	H = 151.55<br>p = 0.000<br>df = 6
Sternite	H = 151.55<br>p = 0.000<br>df = 6	H = 147.81<br>p = 0.000<br>df = 6	H = 149.56<br>p = 0.000<br>df = 6	H = 150.55<br>p = 0.000<br>df = 6	H = 149.21<br>p = 0.000<br>df = 6	H = 149.12<br>p = 0.000<br>df = 6	H = 149.91<br>p = 0.000<br>df = 6	H = 151.56<br>p = 0.000<br>df = 6

H—value of statistics for the Kruskal–Wallis test; p—probability value; df—number of degrees of freedom.

Table S7. Effect of age on urea and uric acid concentrations in the hemolymph and fat body of *A. mellifera* L. workers.

Tissue/location	**urea**	**urea acid**
Hemolymph	H = 187.16<br>p = 0.000<br>df = 2	H = 101.30<br>p = 0.000<br>df = 2
Tergite 3	H = 164.48<br>p = 0.000<br>df = 2	H = 239.95<br>p = 0.000<br>df = 2
Tergite 5	H = 151.16<br>p = 0.000<br>df = 2	H = 162.29<br>p = 0.000<br>df = 2
Sternite	H = 145.21<br>p = 0.000<br>df = 2	H = 138.85<br>p = 0.000<br>df = 2

H—value of statistics for the Kruskal–Wallis test; p—probability value; df—number of degrees of freedom

Table S8. Effect of tissue/location (hemolymph and fat body) on urea and uric acid concentrations in 7-day-old and 14-day-old *A. mellifera* L. workers from different dietary groups.

Groups	7-day-old workers		14-day-old workers	
	**urea**	**urea acid**	**urea**	**urea acid**
Control gr.	H = 82.21<br>p = 0.000<br>df = 3	H = 89.07<br>p = 0.000<br>df = 3	H = 89.08<br>p = 0.000<br>df = 3	H = 80.29<br>p = 0.000<br>df = 3
Hazel	H = 88,72<br>p = 0.000<br>df = 3	H = 89,07<br>p = 0.000<br>df = 3	H = 89,09<br>p = 0.000<br>df = 3	H = 89,07<br>p = 0.000<br>df = 3
Pine	H = 84,13<br>p = 0.000<br>df = 3	H = 89,07<br>p = 0.000<br>df = 3	H = 89,08<br>p = 0.000<br>df = 3	H = 89,07<br>p = 0.000<br>df = 3
Rape	H = 42,95<br>p = 0.000<br>df = 3	H = 39.69<br>p = 0.000<br>df = 3	H = 44.13<br>p = 0.000<br>df = 3	H = 41.20<br>p = 0.000<br>df = 3
Phacelia	H = 89.09<br>p = 0.000<br>df = 3	H = 81.09<br>p = 0.000<br>df = 3	H = 87.66<br>p = 0.000<br>df = 3	H = 89.07<br>p = 0.000<br>df = 3
Goldenrod	H = 89.09<br>p = 0.000<br>df = 3	H = 89.08<br>p = 0.000<br>df = 3	H = 89.09<br>p = 0.000<br>df = 3	H = 89.07<br>p = 0.000<br>df = 3
Buckwheat	H = 87.56<br>p = 0.000<br>df = 3	H = 89.08<br>p = 0.000<br>df = 3	H = 89.08<br>p = 0.000<br>df = 3	H = 80.16<br>p = 0.000<br>df = 3

H—value of statistics for the Kruskal–Wallis test; p—probability value; df—number of degrees of freedom.

Table S9. Effect of diet on urea and uric acid concentrations in the hemolymph and fat body of 7-day-old and 14-day-old *A. mellifera* L. workers.

Tissue/location	7-day-old workers		14-day-old workers	
	**urea**	**urea acid**	**urea**	**urea acid**
Hemolymph	H = 139.35<br>p = 0.000<br>df = 6	H = 130.34<br>p = 0.000<br>df = 6	H = 146.53<br>p = 0.000<br>df = 6	H = 144.39<br>p = 0.000<br>df = 6
Tergite 3	H = 140,65<br>p = 0.000<br>df = 6	H = 140,58<br>p = 0.000<br>df = 6	H = 141,82<br>p = 0.000<br>df = 6	H = 134,95<br>p = 0.000<br>df = 6
Tergite 5	H = 144,22<br>p = 0.000<br>df = 6	H = 146,93<br>p = 0.000<br>df = 6	H = 140,63<br>p = 0.000<br>df = 6	H = 147,00<br>p = 0.000<br>df = 6
Sternite	H = 150,08<br>p = 0.000<br>df = 6	H = 139.10<br>p = 0.000<br>df = 6	H = 148.25<br>p = 0.000<br>df = 6	H = 147.17<br>p = 0.000<br>df = 6

H—value of statistics for the Kruskal–Wallis test; p—probability value; df—number of degrees of freedom.

Table S10. Effect of age on Mg, Ca, and P concentrations in different tissues/locations of *A. mellifera* L. workers.

Tissue/location	**Ca**	**Mg**	**P**
Hemolymph	H = 10.27<br>p = 0.000<br>df = 2	H = 60.92<br>p = 0.000<br>df = 2	H = 4.51<br>p = 0.104<br>df = 2
Tergite 3	H = 10.73<br>p = 0.004<br>df = 2	H = 105.35<br>p = 0.000<br>df = 2	H = 89.91<br>p = 0.000<br>df = 2
Tergite 5	H = 28.66<br>p = 0.000<br>df = 2	H = 211.40<br>p = 0.000<br>df = 2	H = 64.06<br>p = 0.000<br>df = 2
Sternite	H = 27.50<br>p = 0.000<br>df = 2	H = 93.80<br>p = 0.000<br>df = 2	H = 279.43<br>p = 0.000<br>df = 2

H—value of statistics for the Kruskal–Wallis test; p—probability value; df—number of degrees of freedom.

Table S11. Effect of diet on hemolymph Mg, Ca, and P concentrations in 7-day-old and 14-day-old *A. mellifera* L. workers from different dietary groups.

Groups	7-day-old workers			14-day-old workers		
	**Ca**	**Mg**	**P**	**Ca**	**Mg**	**P**
Control gr.	H = 56.49<br>p = 0.000<br>df = 3	H = 23.73<br>p = 0.000<br>df = 3	H = 62.48<br>p = 0.000<br>df = 3	H = 89.19<br>p = 0.000<br>df = 3	H = 68.78<br>p = 0.000<br>df = 3	H = 89.23<br>p = 0.000<br>df = 3
Hazel	H = 39.99<br>p = 0.000<br>df = 3	H = 20.22<br>p = 0.000<br>df = 3	H = 62.21<br>p = 0.000<br>df = 3	H = 84.25<br>p = 0.000<br>df = 3	H = 45.36<br>p = 0.000<br>df = 3	H = 81.09<br>p = 0.000<br>df = 3
Pine	H = 84.27<br>p = 0.000<br>df = 3	H = 79.16<br>p = 0.000<br>df = 3	H = 12.23<br>p = 0.006<br>df = 3	H = 89.14<br>p = 0.000<br>df = 3	H = 44.92<br>p = 0.000<br>df = 3	H = 77.42<br>p = 0.000<br>df = 3
Rape	H = 89.23<br>p = 0.000<br>df = 3	H = 81.06<br>p = 0.000<br>df = 3	H = 59.17<br>p = 0.000<br>df = 3	H = 89.27<br>p = 0.000<br>df = 3	H = 64.51<br>p = 0.000<br>df = 3	H = 80.82<br>p = 0.000<br>df = 3
Phacelia	H = 80.64<br>p = 0.000<br>df = 3	H = 71.11<br>p = 0.000<br>df = 3	H = 80.37<br>p = 0.000<br>df = 3	H = 84.58<br>p = 0.000<br>df = 3	H = 61.06<br>p = 0.000<br>df = 3	H = 89.27<br>p = 0.000<br>df = 3
Goldenrod	H = 89.31<br>p = 0.000<br>df = 3	H = 76.04<br>p = 0.000<br>df = 3	H = 85.59<br>p = 0.000<br>df = 3	H = 75.55<br>p = 0.000<br>df = 3	H = 84.11<br>p = 0.000<br>df = 3	H = 63.32<br>p = 0.000<br>df = 3
Buckwheat	H = 85.10<br>p = 0.000<br>df = 3	H = 66.51<br>p = 0.000<br>df = 3	H = 53.12<br>p = 0.000<br>df = 3	H = 84.77<br>p = 0.000<br>df = 3	H = 55.38<br>p = 0.000<br>df = 3	H = 73.42<br>p = 0.000<br>df = 3

H—value of statistics for the Kruskal–Wallis test; p—probability value; df—number of degrees of freedom.

Table S12. Effect of diet on Mg, Ca, and P concentrations in the hemolymph of 7-day-old and 14-day-old *A. mellifera* L. workers.

Tissue/location	7-day-old workers			14-day-old workers		
	**Ca**	**Mg**	**P**	**Ca**	**Mg**	**P**
Hemolymph	H = 123.26<br>p = 0.000<br>df = 6	H = 131.13<br>p = 0.000<br>df = 6	H = 133.53<br>p = 0.000<br>df = 6	H = 135.13<br>p = 0.000<br>df = 6	H = 113.32<br>p = 0.000<br>df = 6	H = 140.51<br>p = 0.000<br>df = 6
Tergite 3	H = 135.75<br>p = 0.000<br>df = 6	H = 116.04<br>p = 0.000<br>df = 6	H = 120.11<br>p = 0.000<br>df = 6	H = 136.46<br>p = 0.000<br>df = 6	H = 92.42<br>p = 0.000<br>df = 6	H = 124.89<br>p = 0.000<br>df = 6
Tergite 5	H = 131.37<br>p = 0.000<br>df = 6	H = 130.05<br>p = 0.000<br>df = 6	H = 125.26<br>p = 0.000<br>df = 6	H = 132.63<br>p = 0.000<br>df = 6	H = 117.71<br>p = 0.000<br>df = 6	H = 125.49<br>p = 0.000<br>df = 6
Sternite	H = 143.24<br>p = 0.000<br>df = 6	H = 119.96<br>p = 0.000<br>df = 6	H = 130.18<br>p = 0.000<br>df = 6	H = 141.57<br>p = 0.000<br>df = 6	H = 107.12<br>p = 0.000<br>df = 6	H = 125.27<br>p = 0.000<br>df = 6

H—value of statistics for the Kruskal–Wallis test; p—probability value; df—number of degrees of freedom.
